# Lisinopril prevents bullous pemphigoid induced by dipeptidyl peptidase 4 inhibitors *via* the Mas receptor pathway

**DOI:** 10.3389/fimmu.2022.1084960

**Published:** 2023-01-05

**Authors:** Keisuke Nozawa, Takahide Suzuki, Gen Kayanuma, Hiroki Yamamoto, Kazuki Nagayasu, Hisashi Shirakawa, Shuji Kaneko

**Affiliations:** ^1^ Department of Molecular Pharmacology, Graduate School and Faculty of Pharmaceutical Sciences, Kyoto University, Kyoto, Japan; ^2^ Biological/Pharmacological Research Laboratories, Central Pharmaceutical Research Institute, Japan Tobacco Inc., Osaka, Japan

**Keywords:** bullous pemphigoid, dipeptidyl peptidase 4 inhibitor, lisinopril, Mas receptor, FDA Adverse Event Reporting System, IBM^®^ MarketScan^®^ Research databases, macrophage, matrix metallopeptidase 9

## Abstract

Recent studies have suggested that dipeptidyl peptidase 4 (DPP4) inhibitors increase the risk of development of bullous pemphigoid (BP), which is the most common autoimmune blistering skin disease; however, the associated mechanisms remain unclear, and thus far, no therapeutic targets responsible for drug-induced BP have been identified. Therefore, we used clinical data mining to identify candidate drugs that can suppress DPP4 inhibitor-associated BP, and we experimentally examined the underlying molecular mechanisms using human peripheral blood mononuclear cells (hPBMCs). A search of the US Food and Drug Administration Adverse Event Reporting System and the IBM^®^ MarketScan^®^ Research databases indicated that DPP4 inhibitors increased the risk of BP, and that the concomitant use of lisinopril, an angiotensin-converting enzyme inhibitor, significantly decreased the incidence of BP in patients receiving DPP4 inhibitors. Additionally, *in vitro* experiments with hPBMCs showed that DPP4 inhibitors upregulated mRNA expression of *MMP9* and *ACE2*, which are responsible for the pathophysiology of BP in monocytes/macrophages. Furthermore, lisinopril and Mas receptor (MasR) inhibitors suppressed DPP4 inhibitor-induced upregulation of MMP9. These findings suggest that the modulation of the renin-angiotensin system, especially the angiotensin1-7/MasR axis, is a therapeutic target in DPP4 inhibitor-associated BP.

## 1 Introduction

Bullous pemphigoid (BP) is the most common subepidermal autoimmune blistering disease, characterized by the production of autoantibodies directed against two hemidesmosomal proteins, i.e., BP antigen 180 and BP antigen 230. Several disorders, including autoimmune, neurological, and cardiovascular diseases, as well as neoplasms are associated with BP onset (1), and over 50 drugs were reported to elicit drug-induced BP (2).

DPP4 inhibitors, which are oral antidiabetic agents for treating type 2 diabetes mellitus, were suggested to increase the risk of BP (3). These DPP4 inhibitors not only inhibit the degradation of incretin peptides, which decrease blood glucose levels, but also modulate immune cell functions (4–6), and immune modulation by DPP4 inhibitors is associated with increased risk of BP (6). However, thus far, the underlying mechanisms remain unclear.

Analyzing clinical big data followed by *in vitro* and *in vivo* experimental validation is a valid approach for clarifying the mechanism underlying the occurrence of adverse events and for identifying effective treatments. In previous studies, data obtained from the FDA Adverse Event Reporting System (FAERS) database, which is the largest self-reported adverse event database, were analyzed to identify concomitant drugs that reduce the incidence of adverse events (7, 8). Furthermore, the causal relationships between concomitant drug use and incidence of adverse events have also been demonstrated using insurance claims databases (9, 10).

In this study, we first explored the FAERS and IBM^®^ MarketScan^®^ Research (MarketScan) databases to determine drugs that reduce the incidence of BP associated with the use of DPP4 inhibitors. Subsequently, we examined how the identified drugs would ameliorate BP pathophysiology to determine its underlying therapeutic targets and mechanisms through *in vitro* experiments.

## 2 Materials and methods

### 2.1 FAERS database analyses

Adverse event reports corresponding to the 2004–2019 period were obtained from the FDA website (11). Duplicate reports (comprising 13,632,002 cases) were eliminated, as previously described (12), and the remaining 11,438,031 reports were analyzed. Arbitrary drug names, including trade names and abbreviations, were manually annotated to unified generic names using the Medical Subject Headings descriptor ID. Next, reports of BP were defined using the preferred terms “pemphigoid” in MedDRA (version 23.0). FAERS data analysis was performed as previously described (8) and **Supplemental Table 1**. Volcano plots were produced, and *Z* scores were used instead of *P* values to save space.

### 2.2 MarketScan database analyses

The IBM MarketScan Commercial and Medicare Supplemental databases corresponding to the period from January 2017 to December 2019 were purchased from IBM^®^ Watson Health^®^. The dataset comprised data of 43,723,094 employees, dependents, and retirees in the United States, with primary or Medicare supplemental coverage. The outpatients’ data including the year of birth and sex was extracted (36,399,469 cases). Then, those who received formulations belonging to ATC codes A10BH and C09AA03 were defined as patients prescribed DPP4 inhibitors and lisinopril, respectively (**Supplemental Table 2**). BP cases were defined according to the ICD10 system, based on BP-related symptoms (**Supplemental Table 3**). To produce a time-series analysis of MarketScan data, the software packages “survival” (13) and “MatchIt” (14) in R software v4.1.2 and RStudio (2022.07.1 Build 554 software, R Foundation for Statistical Computing) were used as previously described (9). The incidence of BP associated with the use of DPP4 inhibitors was first evaluated *via* Poisson regression analysis, and the obtained results were expressed as incidence rate ratio (IRR), along with the 95% confidence interval (CI) and *Z* scores. Patients who received any DPP4 inhibitor were categorized into two groups (with and without lisinopril), and 1:1 propensity score matching was performed to eliminate deflections in the number of patients with risk factors. Thereafter, propensity score-matched pairs were created by matching two groups using the nearest-neighbor method based on a 0.2-caliper width. The following categories were used as confounding factors: renal failure (N17–19), liver disorder (K70–77), hypertension (I10–15), diabetes (E10–14), heart disease (I05–09, I20–52), cerebrovascular diseases (I60–69, G45, and G46), cancer (C00–D09), neurologic diseases (G20–22, G30–32, F00–03, G35, G40, and G41), autoimmune diseases (M05–09, L40–41, L43, L93, and M32), loop diuretics (ATC codes C03CA01, C03CA02, C03CC01, and C03CA04), and steroids (ATC code H02A). Further, using matched cohort pairs, the daily and cumulative dosages as well as the periods of administration of DPP4 inhibitors and lisinopril were quantified and compared. The defined daily dosage of each drug was then determined according to the WHO website (15). Next, the cumulative incidences of BP were compared between cohorts with and without lisinopril use *via* conventional survival analysis, and Kaplan–Meier plots were used to visualize survival curves. Statistical significance was evaluated using log-rank tests, and Cox proportional regression analysis was performed to calculate hazard ratios. The number of at-risk individuals indicated the number of patients who may experience BP onset each month.

Clinical laboratory test results involving hemoglobin A1c (HbA1c) in the IBM MarketScan^®^ Lab database were defined using the Loinc codes “4548-4” (n = 1,388,265) and “17856-6” (n = 51,213). We only used data with the result unit including “OF TOTAL HGB,” “TOTAL HGB,” “A1C,” “OF HGB,” “PERCENT,” “%,” “HB,” “T.HGB,” “% OF TOTAL HGB”, and “OF T.” Further, we eliminated data of cases that were reported as “< 0” or “> 100” in our analysis, and data corresponding to patients who underwent HbA1c testing more than twice (before and after the first prescription of DPP4 inhibitors) were analyzed (n = 8,921). The patients were categorized into two groups (with and without BP), and for each group, a two-tailed Wilcoxon matched-pairs signed rank test was performed to evaluate changes in HbA1c from before to after prescription of DPP4 inhibitors. The differences in HbA1c changes between the two groups were further compared using unpaired t-tests with Welch’s correction. Additionally, the patients (n = 8,921) were divided into two groups (with and without lisinopril treatment), and their HbA1c values were analyzed as described above.

### 2.3 Drugs and reagents

Vildagliptin was purchased from Cayman Chemical (Ann Arbor, MI, USA), sitagliptin from MedChemExpress (Monmouth Junction, NJ, USA), and lisinopril dihydrate and A779 were purchased fromTokyo Chemical Industry Co., Ltd. (Tokyo, Japan). Vildagliptin, sitagliptin, and A779 were dissolved in dimethyl sulfoxide, and lisinopril was dissolved in ultrapure sterile water. Thereafter, each solution was diluted with the buffer used for each respective experiment.

Recombinant human M-CSF was purchased from PeproTech (Rocky Hill, NJ, USA), and phytohemagglutinin (PHA), phorbol-12-myristate-13-acetate (PMA), and ionomycin were purchased from Sigma-Aldrich (St. Louis, MO, USA). Brefeldin A solution was purchased from Thermo Fisher Scientific (Cleveland, OH, USA).

### 2.4 Primary human cell isolation

Deidentified peripheral blood samples were obtained from healthy volunteers at Japan Tobacco, Inc., who had not received any drugs for the last 7 days and provided written informed consent to participate in the study. For the isolation of human peripheral blood mononuclear cells (hPBMCs), whole blood samples were diluted with sterile PBS in a 1:1 ratio, overlaid on Lymphoprep^TM^ (Axis-Shield PoCAS, Oslo, Norway) in SepMete-50^TM^ (VERITAS Corporation, Tokyo, Japan), and were centrifuged at 1,200 × *g* and 25°C for 10 min. Thereafter, the top layer containing hPBMCs was collected and washed twice with PBS. Human monocytes were isolated from the hPBMCs using a Human Pan Monocyte Isolation kit (Miltenyi Biotec Inc., Auburn, CA, USA) according to the manufacturer’s instructions. hPBMCs and monocytes were washed and suspended in RPMI-1640 (Wako, Osaka, Japan) supplemented with 10% heat-inactivated FBS (Sigma-Aldrich) and 1% penicillin/streptomycin (Gibco, Carlsbad, CA, USA); thereafter, the cells were plated as indicated below.

### 2.5 Flow cytometry analysis

hPBMCs (5 × 10^5^ cells/well) were cultured in the presence or absence of each compound. Following preincubation with the respective compound for 30 min at 37°C under 5% CO_2_, the cells were stimulated by adding PHA (final concentration 5 μg/mL) and were incubated for 48 h at 37°C under 5% CO_2_. For surface phenotyping, the cells were washed in 2 mM EDTA-PBS, centrifuged at 400 × *g* for 1 min, and resuspended in 50 μL Human BD Fc Block™ (BD Biosciences, Franklin Lakes, NJ, USA) diluted to 1:200 using 2 mM EDTA-PBS for 15 min at 4°C, and were then washed with 2 mM EDTA-PBS. Staining was performed using Brilliant Stain Buffer (BD Biosciences) with combinations of monoclonal antibodies (**Supplemental Table 4**) with Fixable Viability Dye eFluor 780 (Thermo Fisher Scientific) for 20 min at 4°C. Thereafter, the cells were washed with Stain Buffer (BD Biosciences) and fixed with fixation buffer (BD Biosciences). Next, the samples were washed and resuspended in staining buffer. Specifically, for intracellular staining, the cells were centrifuged at 400 × *g* for 1 min and were resuspended in 50 ng/mL PMA, 1 μg/mL ionomycin, and 3 μg/mL brefeldin A solution diluted in RPMI-1640 with 10% FBS. After incubation at 37°C for 3 h under 5% CO_2_, the cells were washed and resuspended in 50 μL Human BD Fc Block diluted to 1:200 with FACS buffer (PBS with 2 mM EDTA and 5% FBS) for 15 min at 4°C. Each sample was then fixed and permeabilized using Foxp3/Transcription Factor Fixation/Permeabilization Concentrate and Diluent (Thermo Fisher Scientific). This was followed by staining using V500 anti-human CD3 at 1:50 (clone: UCHT1, 561416; BD Biosciences), BV421 anti-human CD4 at 1:200 (clone: OKT4, 317434; BioLegend, San Diego, CA, USA), APC anti-human IL17A at 1:100 (clone: BL168, 512334, BioLegend), and PE anti-Foxp3 at 1:100 (clone: 236A/E7, 12-4777-42, Thermo Fisher Scientific) for 20 min at 4°C. The samples were then washed and resuspended in a permeabilization buffer. Finally, the cells were subjected to flow cytometry and were analyzed using a BD LSRFortessa™ X-20 system (BD Biosciences). Data were collected using FACSDiva software (BD Biosciences) and were analyzed using FlowJo 10.8.0 (TreeStar, Ashland, OR, USA).

### 2.6 Quantitative RT-PCR analysis

Monocytes (1 × 10^4^ cells/well) isolated from PBMCs using a MACS system were cultured in the presence or absence of each compound. Following preincubation with the compounds for 30 min at 37°C under 5% CO_2_, the cells were stimulated with or without 50 ng/mL M-CSF and were cultured for seven days at 37°C under 5% CO_2_. Total RNA was isolated from the cells using an RNeasy 96-well Kit (Qiagen, Hilden, Germany) according to the manufacturer’s instructions, and quantitative RT-PCR was performed using a QuantStudio 7 Flex system (Thermo Fisher Scientific) with a TaqMan^®^ RNA-to-Ct 1-Step Kit (Thermo Fisher Scientific) and a TaqMan probe (Thermo Fisher Scientific; human MMP9, Hs00957562_m1; human CD163; Hs00174705_m1, human TNF Hs00174128_m1; human ACE2, Hs01085333_m1; human ACE, Hs00174179_m1; human DPP4, Hs00897386_m1; human MasR, Hs00267157_s1; and human GAPDH, Hs02786624_g1). PCR was conducted after reverse transcription at 48°C for 15 min and activation of AmpliTaq Gold DNA polymerase at 95°C for 10 min, followed by 45 cycles of 95°C for 15 s and 60°C for 1 min. To determine the Ct values, data were analyzed using the QuantStudio 6 and 7 Flex Real-Time PCR System software version 1.2 (Thermo Fisher Scientific). RNA expression values were then normalized to the expression level of the housekeeping gene (GAPDH), were calculated using the ΔΔCt method. Results are reported as expression levels relative to the control or M-CSF stimulation samples.

### 2.7 Statistical analyses

Statistical analyses of FAERS and MarketScan data were performed using R software version 4.1.2, RStudio (2022.07.1 Build 554 software, R Foundation for Statistical Computing), and Graphpad Prism for Windows version 6.07 (GraphPad Software Inc., San Diego, CA, USA). The R package “survival” was used to perform time-series analysis and to compare cumulative incidence, the log-rank test was used. The R package “MatchIt” was used to perform 1:1 propensity score matching and to compare population characteristics, and Chi-square tests were performed. Statistical analyses of changes in HbA1cs were performed using two-tailed Wilcoxon matched-pairs signed rank test and unpaired *t*-test with Welch’s correction. One-way analysis of variance and Tukey’s multiple comparison tests were used to analyze data collected following *in vitro* experiments, using GraphPad Prism software for Windows version 6.07 (GraphPad Software). Statistical significance is reported at *p* < 0.05.

## 3 Results

### 3.1 Adverse event self-reports showed an increased risk of BP following the use of DPP4 inhibitors

**Figure 1:**
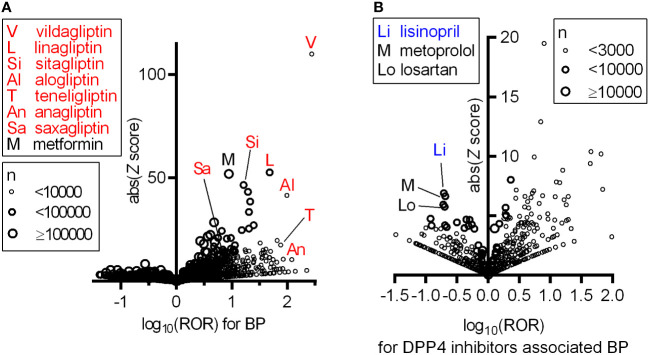
Increased ROR of BP with the use of DPP4 inhibitors and confounding effect of concomitant drugs on BP associated with DPP4 inhibitors based on FAERS data. In these volcano plots, RORs (log scale) and absolute scores are shown. Each circle indicates an individual drug, and circle size reflects the number of patients receiving the drug. **(A)** All DPP4 inhibitors appearing in the FAERS data showing strong and significant increases in the ROR of BP. **(B)** Confounding effects of concomitantly used drugs on the ROR of BP within the population prescribed any of the seven DPP4 inhibitors.

First, we investigated the association between the use of a given drug and the incidence of BP based on FAERS data by calculating the reporting odds ratio (ROR) and *Z* scores during disproportionality analyses (**Supplemental Table 1** BP_DrugA). We observed a class of DPP4 inhibitors that showed a strong association with BP emergence and high RORs and *Z* scores (**Figure 1A**). We selected all DPP4 inhibitors that occurred in FAERS data, i.e., vildagliptin, linagliptin, sitagliptin, alogliptin, teneligliptin, anagliptin, and saxagliptin, for further analyses, and metformin, frequently used with DPP4 inhibitors, exhibited high ROR and *Z* score. Additionally, 18 drugs, categorized as ‘likely’ or ‘probably’ associated with the risk of BP incidence in a previous review (16), showed statistically significant RORs (**Table 1**). After excluding patients who received concurrent metformin or any of the 18 risk drugs, we calculated the RORs of DPP4 inhibitors, which remained significant (**Table 2**). When the patients were stratified by age and sex, BP incidence increased in all categories. Furthermore, the elderly population (aged ≥ 80 years) showed a higher odds ratio, as also observed in a previous study (17).

**Table 1:** List of BP-risk drugs categorized as ‘likely’ or ‘probable’ association with BP in a previous study (16) showing a significant increase in ROR for BP in our analysis, as indicated in **Supplemental Table 1** BP_DrugA.

Likely association		Probable association	
Drug name	ROR (95% CI)	Drug name	ROR (95% CI)
Aspirin	2.7 (2.4–3.0)	Atezolizumab	3.7 (1.7–7.7)
Furosemide	4.8 (4.3–5.3)	Bumetanide	6.2 (4.3–9.0)
Nivolumab	19.5 (17.0–22.3)	Cephalexin	4.3 (3.0–6.1)
Pembrolizumab	15.0 (12.1–18.6)	Diclofenac	1.6 (1.2–2.2)
		Durvalumab	7.6 (3.4–16.8)
		Fluoxetine	1.5 (1.1–2.0)
		Griseofulvin	23.1 (5.7–92.7)
		Hydrochlorothiazide	4.1 (3.6–4.6)
		Ipilimumab	7.4 (5.5–10.0)
		Losartan	2.0 (1.6–2.4)
		Rosuvastatin	1.9 (1.5–2.4)
		Spironolactone	4.7 (3.9–5.6)
		Terbinafine	2.4 (1.2–4.9)
		Ustekinumab	2.0 (1.3–2.9)

**Table 2:** Relationship between the emergence of bullous pemphigoid (BP) and the prescription of DPP4 inhibitors (DPP4i) based on FAERS data. (+); patients receiving DPP4 inhibitors or exhibiting BP; (–); patients not receiving DPP4 inhibitors or not exhibiting BP.

Group		DPP4i (+) BP (+)	DPP4i (+) BP (–)	DPP4i (–) BP (+)	DPP4i (–) BP (–)	ROR	RORlower	ROR upper	*Z* score
All patients		1,075	103,693	2,359	11,330,904	49.8	46.3	53.5	105.8
After the exclusion of at-risk drug users		271	35,581	1,294	9,776,664	57.5	50.5	65.6	60.5
Stratified by sex and age									
Male	< 80 years	331	30,866	731	2,376,389	34.9	30.6	39.7	53.4
	≥ 80 years	216	4,582	248	240,515	45.7	38.0	55.0	40.6
Female	< 80 years	197	28,944	630	3,742,545	40.4	34.4	47.5	45.2
	≥ 80 years	196	5,085	290	352,365	46.8	39.0	56.3	41.1

### 3.2 Based on FAERS data, lisinopril reduced the risk of DPP4 inhibitor-induced BP

We searched for concomitant drugs that reduced the RORs of BP in patients receiving DPP4 inhibitors using FAERS data. Thus, several concomitant drugs, including lisinopril, metoprolol and losartan, that could decrease the ROR of BP in patients treated with DPP4 inhibitors were identified (**Figure 1B** and **Supplemental Table 1** DPP4iBP_DrugB). Among these drugs, lisinopril, which by itself did not affect BP risk, showed the highest absolute *Z* score. The other identified angiotensin (Ang)-converting enzyme (ACE) inhibitors did not have a significant effect on DPP4 inhibitor-associated BP.

### 3.3 Based on insurance claims data, increased incidence of BP owing to DPP4 inhibitor use was decreased by concomitant use of lisinopril

**Figure 2:**
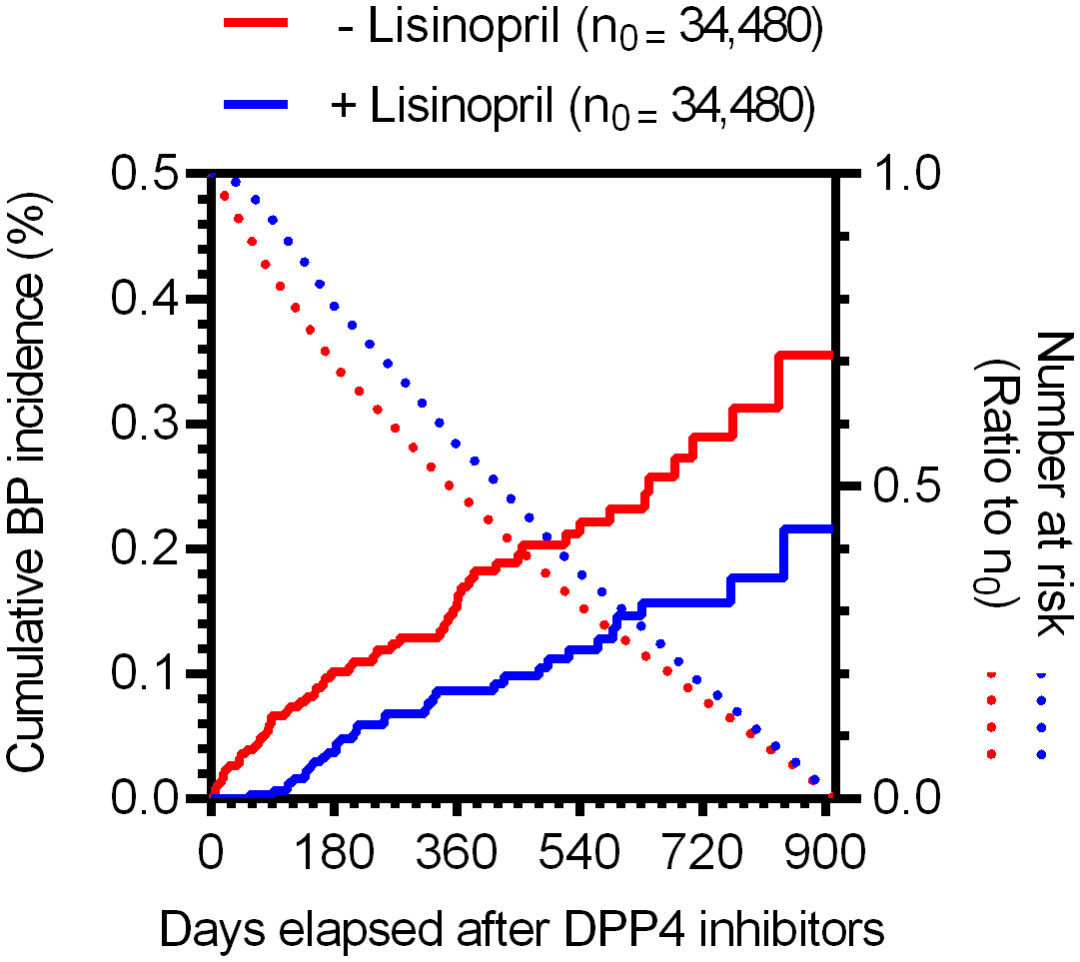
Time trends in the incidence of BP for individuals in the propensity score-matched cohorts receiving DPP4 inhibitors based on MarketScan data. Kaplan–Meier curves of the cumulative incidence ratio of BP in patients receiving DPP4 inhibitors, shown individually for two groups: without (red) and with (blue) lisinopril. Dotted lines indicate the numbers of at-risk patients as a ratio to the initial number of patients (*n*_0_ = 34,480, for each group).

To examine the causal relationship between the use of DPP4 inhibitors and/or lisinopril and BP onset, we analyzed MarketScan data. The ingredients of the DPP4 inhibitor and lisinopril formulations are shown in **Supplemental Table 2**. Further, BP cases were defined based on BP-related symptoms, as listed in **Supplemental Table 3**. Therefore, considering the time distribution of the first event after enrolment in the MarketScan database, the number of patients who were initially prescribed DPP4 inhibitors or lisinopril was markedly higher during the first six months and plateaued after seven months (**Supplemental Figure 1**), indicating that patients prescribed DPP4 inhibitors or lisinopril within six months after enrolment may have used these drugs before enrolment. We thus excluded patients who received a diagnosis of BP or a prescription of DPP4 inhibitors or lisinopril during the 0- to 180-days run-in period from the study cohort. Thereafter, we evaluated the overall association between the use of DPP4 inhibitors and the onset of BP by estimating the IRR of BP. The DPP4 inhibitor group showed high IRR values (**Table 3**). We also evaluated the effects of changes in blood glucose levels on BP onset, showing that prescription of DPP4 inhibitors reduced HbA1c levels with or without BP onset. However, changes in HbA1c levels did not differ between the groups (**Supplemental Figure 2**), indicating that prescription of DPP4 inhibitors increased the risk of BP, regardless of their blood glucose level-reducing effect. To estimate the effects of lisinopril on BP onset after prescription of DPP4 inhibitors, we defined the concomitant use of lisinopril as the use of lisinopril after prescription of DPP4 inhibitors, and we divided the DPP4 inhibitor cohort into two populations: those who received lisinopril (n = 34,480) and those who did not (n = 78,069). In the next step, we performed 1:1 propensity score matching (**Table 4**) to eliminate known confounding factors associated with the onset of BP and the prescription of DPP4 inhibitors and lisinopril (18, 19). In the matched cohorts, combination with lisinopril showed significantly reduced BP incidence, with a hazard ratio of 0.53 (95% confidence interval [CI]: 0.35–0.81, *p* = 0.003 in the log-rank test; **Figure 2**). Further, in these matched cohorts, daily and cumulative doses as well as the DPP4 inhibitor administration period were equivalent in each pair, with or without lisinopril use (**Table 5**).

**Table 3:** Incidence rate ratio (IRR) of bullous pemphigoid (BP) with the prescription of DPP4 inhibitors based on MarketScan data.

DPP4 inhibitors	BP cases	Incidence rate (% per person-year)	IRR (95% CI)	*Z* score	–log_10_ *p*
+	179/112,549	0.148	1.68 (1.45–1.95)	6.93	11.4
–	40,874/31,336,175	0.088			

**Table 4:** Propensity score matching of cohorts receiving DPP4 inhibitors in the MarketScan database. The number and percentage of patients in each group are shown.

	Before matching			After matching		
	Without lisinopril(%)	With lisinopril(%)	*P* value	Without lisinopril(%)	With lisinopril(%)	*P* value
Total	78,069	34,480	–	34,480	34,480	–
Elderly (≥ 65 years)	11,053	3,518	< 0.001	3,524	3,518	0.95
	(14.2%)	(10.2%)		(10.2%)	(10.2%)	
Female	37,489	14,078	< 0.001	14,100	14,078	0.87
	(48%)	(40.8%)		(40.9%)	(40.8%)	
Renal failure	9,901	4,260	0.130	4,238	4,260	0.81
	(12.7%)	(12.4%)		(12.3%)	(12.4%)	
Liver disorder	10,859	4,432	< 0.001	4,426	4,432	0.95
	(13.9%)	(12.9%)		(12.8%)	(12.9%)	
Hypertension	59,160	31,392	< 0.001	31,389	31,392	0.98
	(75.8%)	(91%)		(91%)	(91%)	
Diabetes	76,414	34,163	< 0.001	34,172	34,163	0.75
	(97.9%)	(99.1%)		(99.1%)	(99.1%)	
Heart diseases	23,037	9,922	0.013	9,920	9,922	0.99
	(29.5%)	(28.8%)		(28.8%)	(28.8%)	
Cerebrovascular diseases	7,230	2,822	< 0.001	2,816	2,822	0.94
	(9.3%)	(8.2%)		(8.2%)	(8.2%)	
Cancer	9,021	3,379	< 0.001	3,380	3,379	1.00
	(11.6%)	(9.8%)		(9.8%)	(9.8%)	
Neurologic diseases	2,975	1,120	< 0.001	1,076	1,120	0.35
	(3.8%)	(3.2%)		(3.1%)	(3.2%)	
Autoimmune diseases	4,088	1,473	< 0.001	1,442	1,473	0.57
	(5.2%)	(4.3%)		(4.2%)	(4.3%)	
Loop diuretics	8,632	3,314	< 0.001	3,297	3,314	0.84
	(11.1%)	(9.6%)		(9.6%)	(9.6%)	
Steroids	26,043	10,750	< 0.001	10,753	10,750	0.99
	(33.4%)	(31.2%)		(31.2%)	(31.2%)	

**Table 5:** Daily and cumulative doses and administration periods of DPP4 inhibitors and lisinopril in the propensity score-matched cohorts.

	Without lisinopril		With lisinopril			
	DPP4 inhibitor		DPP4 inhibitor		Lisinopril	
	Median(IQR)	Range	Median(IQR)	Range	Median(IQR)	Range
Average daily dose (DDD*)	1 (1–1)	0.001 –1000	1 (1–1)	0.005 –1000	1.5 (0.86–2)	0.05 –2000
Cumulative dose (DDD*)	180 (90–360)	0.12 – 630000	180 (90–390)	0.15 –390000	360 (125–810)	1 –720000
Administration period (day)	180 (90–390)	1 –1440	210 (90–420)	1 –1515	270 (120–450)	1 –1,740

The median value, interquartile range (IQR), and minimum-maximum ranges are shown for each group. Each dose is shown as a ratio to the defined daily dose (DDD). *DDD: 100 mg sitagliptin, 5 mg saxagliptin, 25 mg alogliptin, 5 mg linagliptin, or 10 mg lisinopril was considered as 1.

### 3.4 Lisinopril suppressed matrix metallopeptidase 9 upregulation by vildagliptin in human monocytes/macrophages

**Figure 3:**
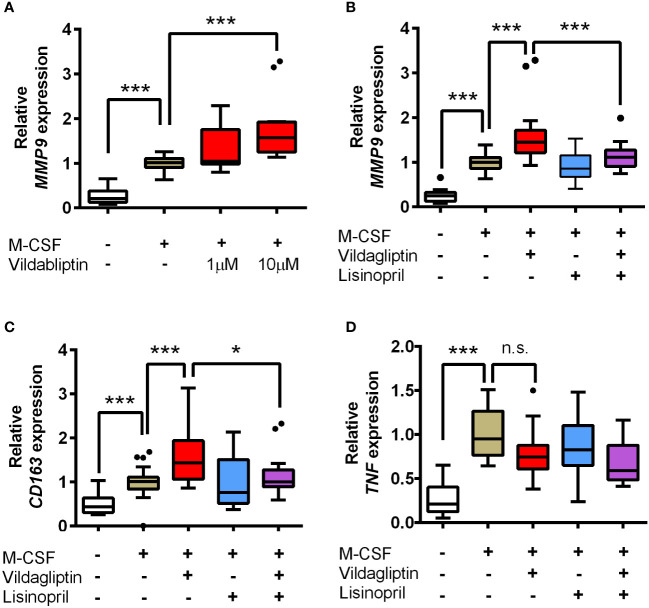
Effects of vildagliptin and lisinopril on the expression levels of *MMP9*, *CD163*, and *TNF* in human monocytes/macrophages stimulated using M-CSF. Monocytes were isolated from hPBMCs using the MACS system and were cultured for seven days in the presence or absence of each compound with or without 50 ng/mL M-CSF. mRNA levels per gene were assessed in the indicated number (*n*) of donors based on three to six replicates per donor and were normalized to GAPDH. Data are relative to the M-CSF group at a concentration of 1. **(A)** Concentration-dependent effects of vildagliptin (1–10 μmol/L) on *MMP9* expression (*n* = 4). **(B–D)** Combined effect of lisinopril (10 μmol/L) and vildagliptin (10 μmol/L) on *MMP9* expression (*n* = 8; ***p < 0.005), *CD163* (*n* = 6; *p < 0.05, ***p < 0.005), and *TNF* (n = 6; ***p < 0.005; n.s., not significant). Boxes indicate the 25th and 75th percentiles; lines inside the boxes indicate medians, and whiskers indicate the upper and lower adjacent values (3/2-fold the interquartile range from the end of the box) as per Tukey’s test. Dots represent outliers beyond the whiskers’ range.

Several studies have suggested that the production of matrix metallopeptidase 9 (MMP9) from immune cells is associated with skin-blister formation during BP (20) and that ACE inhibitors suppress MMP9 expression and inhibit MMP9 activity (21–24). Therefore, we hypothesized that the molecular mechanism underlying the bidirectional effects of DPP4 inhibitors and lisinopril on BP is closely related to the regulation of MMP9 function. It has been reported that monocytes, macrophages, neutrophils, and eosinophils are sources of MMP9 in cases with BP (25–28). However, eosinophil infiltration into the skin is significantly less frequent in BP patients treated with DPP4 inhibitors than that in patients who do not receive DPP4 inhibitors (29). It was also reported that the reduction of skin infiltrating and circulating eosinophils was more pronounced in DPP4 inhibitor-associated BP with a noninflammatory phenotype (30). These data indicated that contribution of eosinophils in the DPP4 inhibitors received BP patients with a noninflammatory phenotype might less than in the idiopathic BP patients. In addition, monocytes enhance neutrophil-induced blister formation in *ex vivo* model (26). Hence, we focused on MMP9 production by monocytes and macrophages using hPBMCs. In the experiment, monocytes from healthy volunteers were stimulated using M-CSF for seven days, with or without vildagliptin, which showed the highest ROR, in line with the analyses of FAERS data. We used 1 and 10 μmol/L vildagliptin because its plasma concentration is up to 10 μmol/L in clinical use (31, 32); we observed that the vildagliptin-treated cells showed higher *MMP9* mRNA expression, compared to their untreated counterparts, in a concentration-dependent manner (**Figure 3A**). Further, to determine the effects of DPP4 inhibitor treatment on T cell differentiation, hPBMCs were stimulated with PHA, with or without vildagliptin and sitagliptin, and the proportions of Th1, Th17, and Treg cells were analyzed using flow cytometry. Thereafter, the classification of each T cell subset was determined using cell surface markers (**Supplemental Figures 3, 4**), based on a previous review (33) or a combination of IL-17 and Foxp3 (**Supplemental Figure 5**). However, we did not observe any distinct changes in these cell populations following treatment with vildagliptin or sitagliptin. We subsequently investigated the effect of lisinopril on DPP4 inhibitor-induced MMP9 mRNA changes. We used 10 μM lisinopril, a concentration that is more than 10-fold higher than the plasma concentration recommended for clinical use (34) but does not inhibit MMP9 directly (21), to evaluate the maximum inhibitory effect of lisinopril on ACE. The increase in *MMP9* mRNA expression induced by vildagliptin was reduced by the concomitant of lisinopril treatment; however, using lisinopril only did not affect *MMP9* mRNA levels (**Figure 3B**). To identify the phenotype of these cells, we measured *TNF* (M1 type) and *CD163* (M2 type) mRNA levels, which showed an increase in mRNA levels of the M2 type marker following vildagliptin treatment. We also observed that this increase was reduced by lisinopril combination treatment (**Figure 3C**). mRNA levels of the M1 type marker tended to decrease under the vildagliptin treatment; however, the effect was not significant (**Figure 3D**).

### 3.5 DPP4 inhibitors upregulated MMP9 expression through the Ang1-7/MasR axis

**Figure 4:**
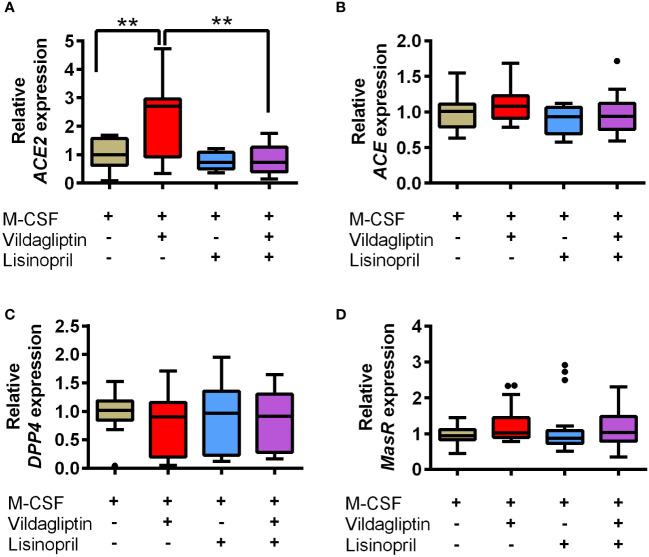
Effects of vildagliptin and lisinopril on the expression of RAS components and DPP4 enzymes in human monocytes/macrophages stimulated using M-CSF. Monocytes were isolated from hPBMCs using the MACS system and cultured for seven days with 50 ng/mL M-CSF in the presence or absence of each compound. The concentration of vildagliptin and lisinopril was 10 μmol/L, each. mRNA levels were assessed in three to six donors with three to six replicates per donor and were normalized using GAPDH as a reference. Data are relative to the M-CSF group at a concentration of 1. The effects of each compound on ACE2 **(A)**, ACE **(B)**, DPP4 **(C)**, and MasR (MAS1) **(D)** expression are shown. Boxes indicate the 25th and 75th percentiles; lines inside the boxes indicate medians, and whiskers indicate the upper and lower adjacent values (3/2-fold the interquartile range from the end of the box) as per Tukey’s test. Dots represent outliers beyond the whiskers’ range **p < 0.01.

**Figure 5:**
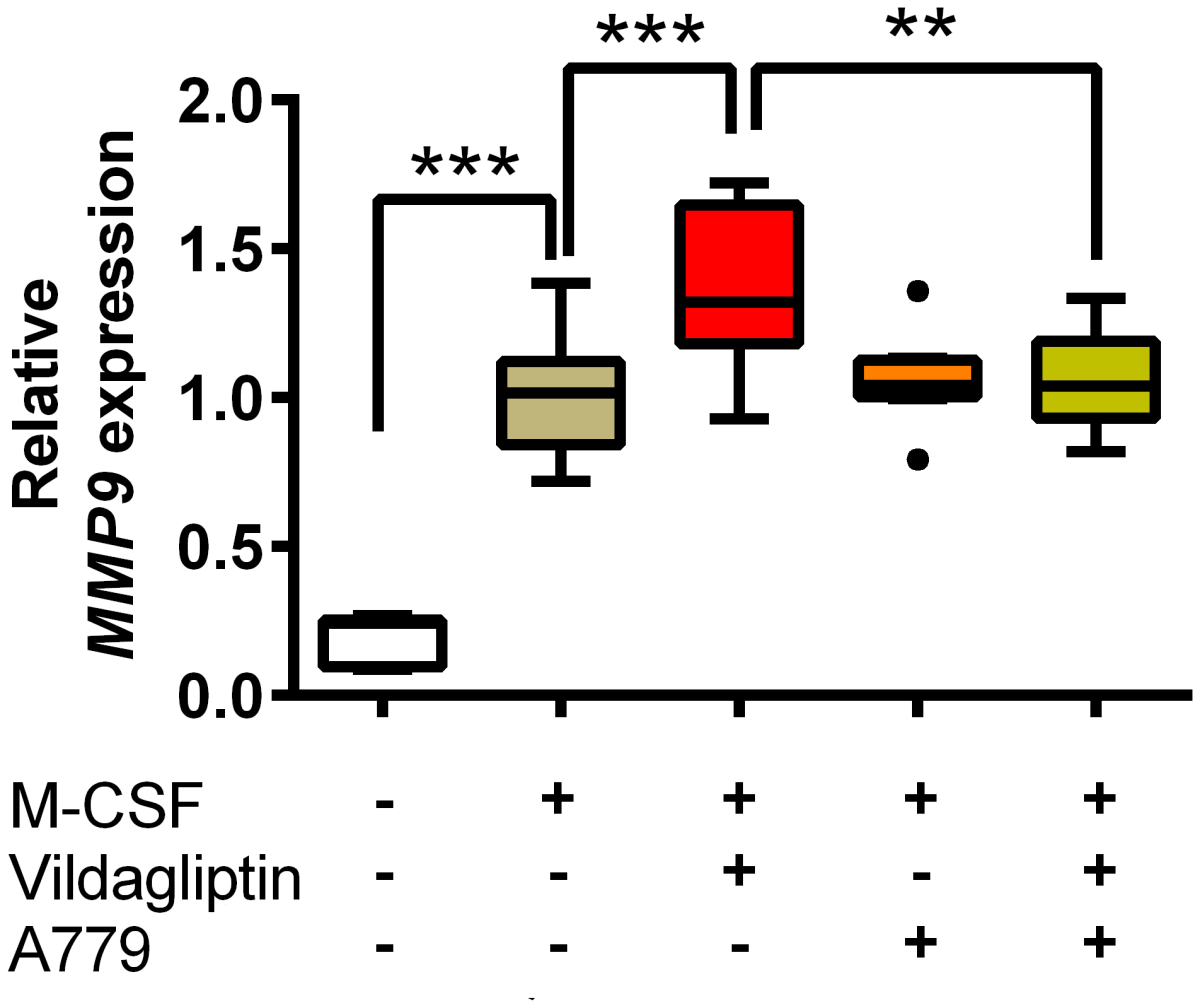
MasR inhibitor inhibited the upregulation of *MMP9* induced by DPP4 inhibitors. Monocytes isolated from hPBMCs were treated with M-CSF and the indicated compounds for seven days. The concentration of vildagliptin and A779 was 10 μmol/L. The expression of MMP9 was measured from three donors with three to six replicates for each donor and was normalized to GAPDH. Data are relative to the M-CSF group at a concentration of 1. Boxes indicate the 25th and 75th percentiles; lines inside the boxes indicate medians, and whiskers indicate the upper and lower adjacent values (3/2-fold the interquartile range from the end of the box) as per Tukey’s test. Dots represent outliers beyond the whiskers’ range **p < 0.01; ***p < 0.005.

ACE and ACE2 are the key enzymes responsible for the generation of several components of the renin-angiotensin system (RAS), including Ang II, Ang III, Ang1-7, and Ang1-9, and RAS components affect various cell responses *via* their receptors. We examined the effects of DPP4 inhibitors on *ACE* and *ACE2* mRNA expression in monocytes/macrophages, which showed that vildagliptin increased *ACE2* mRNA expression, but did not affect *ACE* mRNA levels (**Figures 4A, B**). Further, combination with lisinopril suppressed the upregulation of *ACE2* mRNA by vildagliptin (**Figure 4A**). In contrast, *DPP4* mRNA expression was not affected by either drug (**Figure 4C**), suggesting that *ACE2* mRNA expression plays an important role in enhancing *MMP9* mRNA expression in monocytes/macrophages. Recent studies have suggested that Mas receptor (MasR) and Ang1-7 modulate M1/M2 macrophage polarization (35, 36). Further, based on *in vivo* chronic kidney disease models, DPP4 inhibitors were reported to enhance Ang1-7 production through ACE2 expression (37). We thus evaluated whether DPP4 inhibitors affected the Ang1-7/MasR axis, showing that vildagliptin did not affect *MAS1* (MasR) expression (**Figure 4D**). We then used A779, a MasR inhibitor, to examine the effects of vildagliptin on the Ang1-7/MasR axis, showing that the vildagliptin-induced increase in *MMP9* mRNA levels was suppressed by combination with A779; however, A779-only treatment did not affect *MMP9* mRNA levels (**Figure 5**).

## 4 Discussion

To the best of our knowledge, this study is the first to demonstrate that lisinopril prevents the onset of DPP4 inhibitor-associated BP in patients with type 2 diabetes. This outcome is supported by data mining analyses using two independent sets of clinical big data and *in vitro* experiments using human primary monocytes/macrophages. Our results also suggested that the Ang1-7/MasR axis is the mechanism underlying DPP4 inhibitor-associated BP, based on MMP9 production by monocytes/macrophages. This was also supported by the results of *in vitro* experiments using the MasR inhibitor A779.

Several case reports, pharmacovigilance data analyses, and observational studies have suggested that DPP4 inhibitors may play a role in BP development, as recently confirmed through a meta-analysis of randomized controlled clinical trials (38). However, little is known regarding the underlying mechanisms. Additionally, it is important to explore treatment methods for DPP4 inhibitor-associated BP. Therefore, in the present study, we used adverse event data from the FAERS database, on the basis of which several unexpected drug-drug interactions were identified as confounding factors (7–10). Our analysis in this regard not only suggested that DPP4 inhibitors could cause BP, as indicated by previous studies (39, 40), but also indicated that lisinopril could suppress DPP4 inhibitor-induced BP. Conversely, the anti-PD1/PDL1 antibody group (nivolumab, pembrolizumab, cemiplimab, atezolizumab, avelumab, and durvalumab) showed increased risk of BP (ROR = 16, *Z* score = 48), and combination treatment with lisinopril did not affect their odds ratios (ROR=1.6, *Z* score = 1.2). These observations indicated that the reducing effect of lisinopril on BP onset may be specific to DPP4 inhibitors.

The preventive effect of lisinopril on BP associated with DPP4 inhibitors was confirmed by analyzing MarketScan data to identify potential causal relationships. To extract BP cases from the MarketScan data, we used “bullous pemphigoid” plus other BP-related terms as listed in **Supplemental Table 3**, given that BP is sometimes reported using other similar terms (41). In our analysis, the incidence rate of BP was higher in the DPP4 inhibitor cohort than in the control cohort (0.148% vs. 0.088% per person-years, IRR = 1.68), consistent with the findings of a previous study (42). In propensity score-matched cohorts of DPP4 inhibitors with or without lisinopril, lisinopril showed a significant reducing effect on DPP4 inhibitors associated with BP, with a hazard ratio of 0.53, with no difference in average daily doses, cumulative doses, and the administration period of DPP4 inhibitors. We also confirmed whether changes in HbA1c levels affected BP onset, given that DPP4 inhibitors are antidiabetic agents that are used for type 2 diabetes mellitus treatment owing to their ability to reduce blood glucose levels by inhibiting the degradation of incretin peptides. HbA1c levels did not differ between the BP onset and no BP onset groups, indicating that onset of BP associated with DPP4 inhibitors is independent of blood glucose levels. We also found that lisinopril did not affect HbA1c levels in patients using DPP4 inhibitors, indicating that the mechanism by which the risk of BP onset is reduced by lisinopril is independent of changes in blood glucose levels.

DPP4 is a type II membrane peptidase, and the DPP4 gene family enzymes include DPP8, DPP9, and fibroblast activation proteins. DPP4 inhibitors also inhibit these other DPP4 gene family enzymes, and lowering the relative selectivity between DPP4 and DPP8/9 may result in adverse effects (43). Further, in the FAERS data, seven DPP4 inhibitors, i.e., vildagliptin, linagliptin, sitagliptin, alogliptin, teneligliptin, and saxagliptin, were identified, and we observed a significant association between the use of these compounds and BP incidence, independent of selectivity towards DPP8/9. These results are consistent with those of a previous study based on FAERS data, randomized controlled trials and Meta-analysis (39, 44, 45). These data may indicate that the inhibition of DPP4 enzyme activity increased the incidence of DPP4 inhibitor-induced BP.

Additionally, our analysis based on FAERS data suggested that lisinopril is a candidate drug for suppressing the onset of DPP4 inhibitor-associated BP. However, other ACE inhibitors did not show any significant inhibitory effects in this regard. This may be due to the absence of prescriptions, which possibly affected statistical power. Based on the FAERS database, the total number of cases using lisinopril is approximately 240,000, and with regard to the use of lisinopril in combination with DPP4 inhibitors, it is approximately 8,000; however, the total number of cases using other ACE inhibitors is less than 100,000 (**Supplemental Table 1** BP_DrugA). An alternative explanation would be non-specific effects of lisinopril; however, to the best of our knowledge, there is no evidence that lisinopril functions *via* a different molecular mechanism than other ACE inhibitors. Therefore, further research using other datasets is required to elucidate whether only lisinopril reduces the incidence of DPP4 inhibitor-associated BP.

Several studies have suggested that MMP9 production by monocytes/macrophages contributes to BP onset, especially blister formation (25, 28). The results of the present study also revealed that DPP4 inhibitors promoted this pathophysiology. Specifically, we demonstrated that DPP4 inhibitors promoted M2 marker expression, indicating that the increase in *MMP9* mRNA levels depended on M2 polarization, as demonstrated previously (28). The M1 marker expression tended to decrease in the M-CSF group; however, the change was not statistically significant. One possible explanation for this observation may be the culture conditions used here. M-CSF stimulation was employed because it promotes M2 macrophage differentiation. Further experiments with stimulation by GM-CSF, which promotes M1 polarization, are needed to clarify the effect of DPP4 inhibitors on differentiation to M1 type. Moreover, additional markers and/or flow cytometry analysis to detect cell surface markers should be used to confirm M1/M2 polarization.

Reportedly, DPP4 inhibitors effect Th1 and Th17 differentiation, which may be associated with BP onset (46); however, we did not observe such changes in our experiments. Possibly, this discrepancy was due differences in drug concentrations. In a previous study, sitagliptin at 50 μg/mL (approximately 80 μmol/L), which was higher than the concentration used in the current study and clinical plasma concentration (47), possibly resulted in non-specific effects. For example, sitagliptin inhibits fibroblast activation protein at an IC_50_ of approximately 80 μM (48). Regardless, additional studies under physiological conditions are needed to determine whether DPP4 inhibitors affect T cell differentiation through inhibition of DPP4 enzyme activity.

Our *in vitro* data based on the use of lisinopril supported the hypothesis that DPP4 inhibitors promote BP onset *via* MMP9 production by monocytes/macrophages. We also showed that lisinopril, a candidate drug for BP treatment identified through clinical data mining, suppressed *MMP9* mRNA expression and M2 polarization induced by DPP4 inhibitors. Additionally, using a combination of A779 and vildagliptin indicated that the inhibition of the Ang1-7/MasR axis suppressed *MMP9* mRNA expression. A previous *in vivo* study suggested that DPP4 inhibitors promote Ang1-7 production by accelerating ACE2 expression, but do not change ACE and MasR expression (35). This is consistent with the results of our *in vitro* study. Ang1-7 promotes M2 macrophage polarization *via* MasR (36), and MMP9 production by M2 macrophages is associated with BP pathogenesis, as discussed above. These previous studies and our present results gave rise to the novel hypothesis that the Ang1-7/MasR axis modulates DPP4-associated BP, and this hypothesis is supported by evidence that lisinopril inhibits *ACE2* mRNA expression. In contrast, it has been reported that ACE inhibitors increase Ang1-7 production *in vitro* and *in vivo* (49, 50). We did not detect Ang1-7 in our experiments using ELISA, possibly because its concentrations were below the limit of detection. Therefore, further research focusing on the production of RAS components is needed to reveal whether ACE inhibitors modulate Ang1-7 production by DPP4 inhibitors and to elucidate how lisinopril inhibits *ACE2* mRNA expression.

There are several limitations of the current study. In data mining of the FAERS and MarketScan databases, misclassification of the outcome is a possible limitation because the International Classification of Disease 10 (ICD10)-based code is insufficient for differentiating BP from other bullous diseases (41). Future work on the validation of claims-based algorithms for BP will help define the outcome. The low incidence of BP also hampers the performance of cohort studies. For instance, we were unable to analyze concomitant effects of lisinopril on each DPP4 inhibitor because the number of total events was low. Future analyses of multiple databases may help overcome these issues. A further limitation of this study is that DPP4 inhibitors and lisinopril affect multiple cellular functions, such as immune modulation, *via* their own respective targets. Because of this, other immune cells may also affect BP onset. Further *in vivo* studies to identify the underlying molecular mechanisms may provide valuable insights for addressing this challenge.

In conclusion, our results, based on clinical big data mining followed by experimental validation *in vitro*, demonstrated that lisinopril is effective for reducing the risk of BP associated with the use of DPP4 inhibitors, suggesting that MMP9 and ACE2 expression play important roles in the underlying mechanisms. Further, our results indicated that the Ang1-7/MasR axis is responsible for this mechanism. Therefore, these findings provide new insights into the pathophysiology of BP and also highlight a new drug target for treating BP.

## Data availability statement

The original contributions presented in the study are included in the article/**Supplementary Material**. Further inquiries can be directed to the corresponding author.

## Ethics statement

Human peripheral blood samples were obtained from healthy volunteers who provided written informed consent in accordance with relevant guidelines and regulations. The study protocol was approved by the ethics committee of Japan Tobacco, Inc. (Tokyo, Japan).

## Author contributions

KNo and SK designed the study. KNo, TS, GK, and HY analyzed the clinical data. K Nozawa performed the *in vitro* experiments. KNo, KNa, HS, and SK analyzed the data and wrote the manuscript. All authors contributed to the article and approved the submitted version.
