# Supplementary material for: Lisinopril prevents bullous pemphigoid induced by dipeptidyl peptidase 4 inhibitors via the Mas receptor pathway
Source: Front Immunol. 2023 Jan 5;13:1084960. doi: 10.3389/fimmu.2022.1084960 (PMC9849361; doi:10.3389/fimmu.2022.1084960)
Supplement: Supplementary file 2 [file Presentation_1.pdf]

frontiers

# *Supplementary Material*

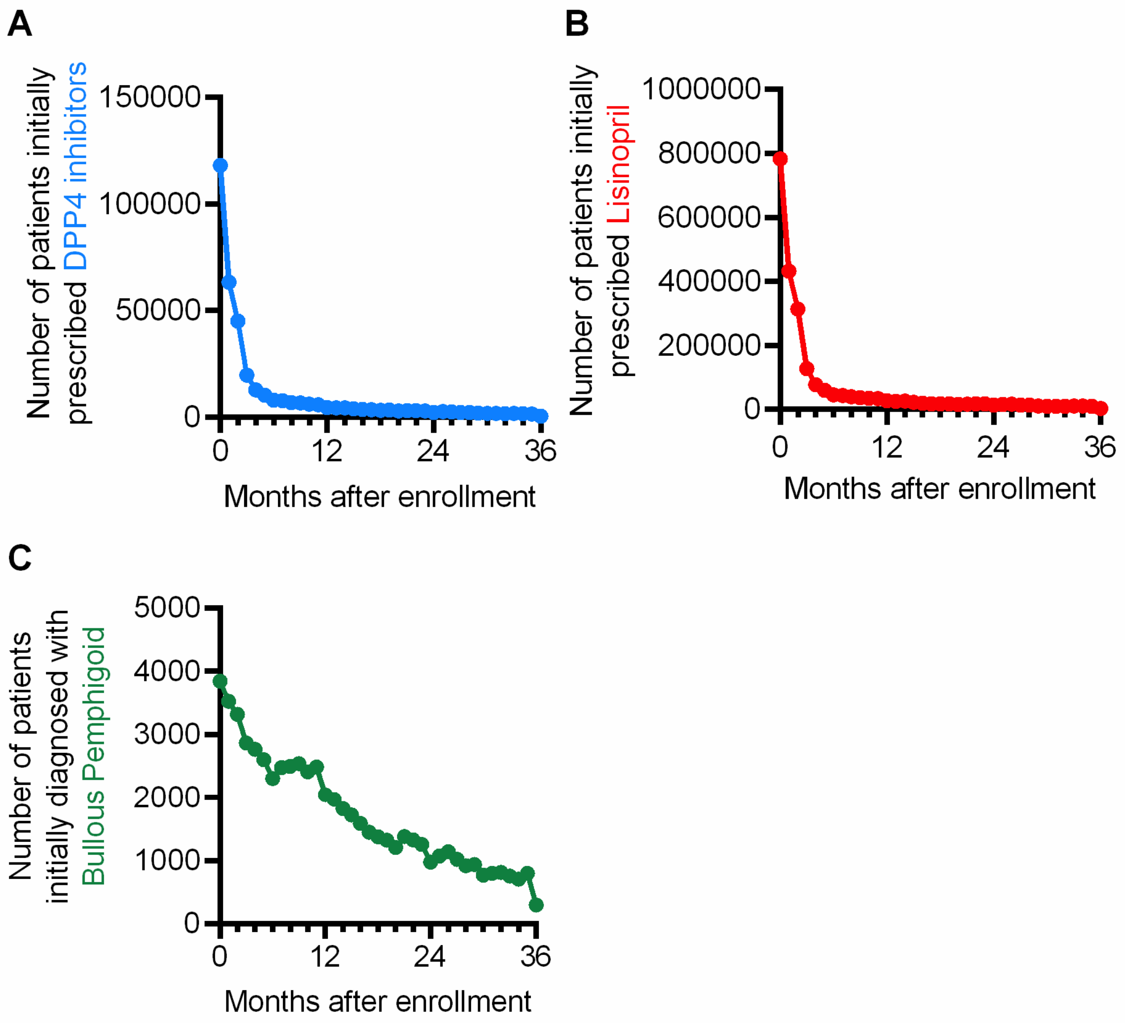


Supplemental Figure 1.

Time distribution of the first event after enrolment in the MarketScan database. (**A**) Time interval from patient enrollment to the first prescription of DPP4 inhibitors. (**B**) Time interval from patient enrollment to the first prescription of lisinopril. (**C**) Time interval from patient enrollment to initial BP diagnosis. The number of patients is presented on a monthly basis.

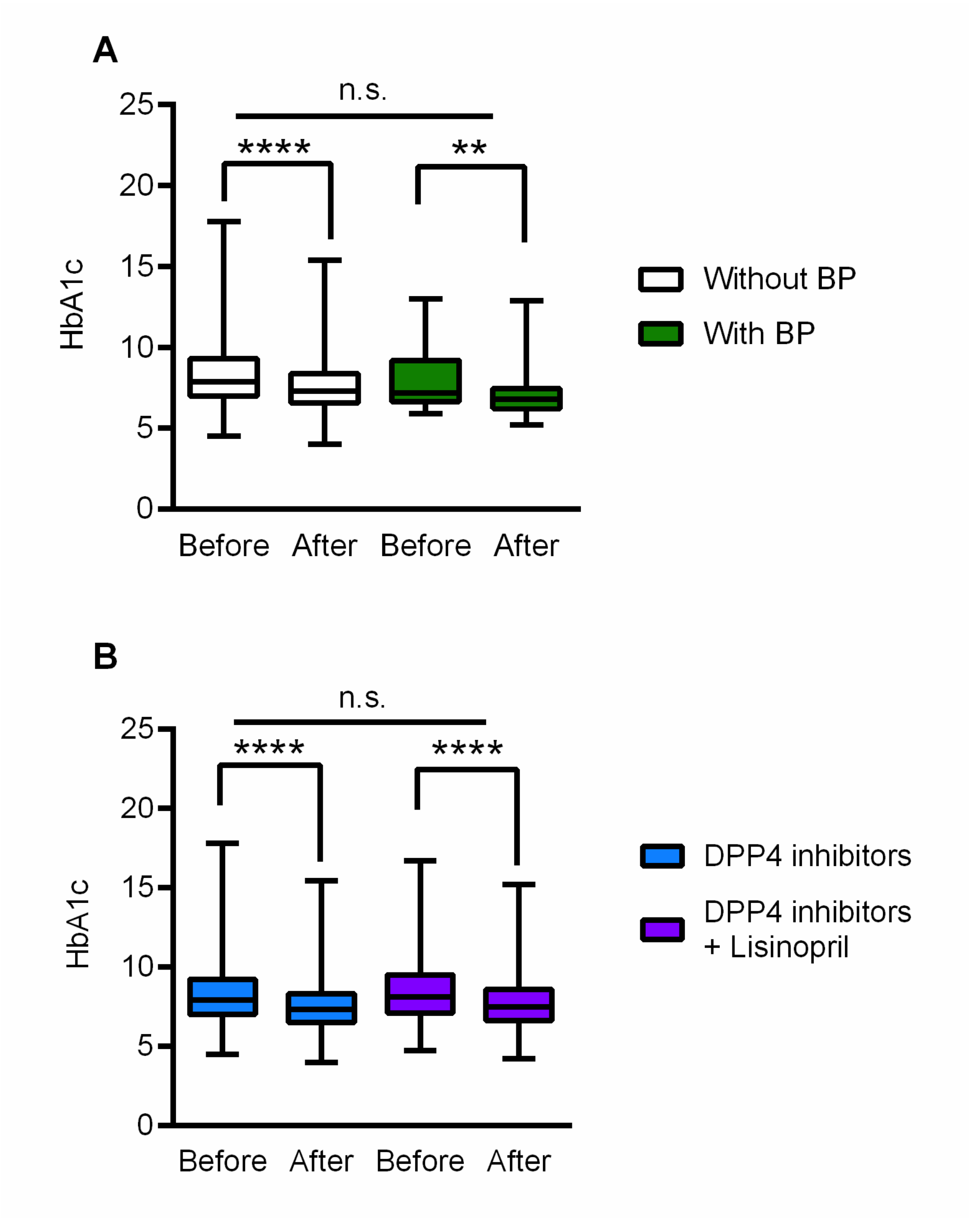


Supplemental Figure 2.

Chronological changes in the HbA1c levels of patients receiving DPP4 inhibitors. HbA1c levels before and after DPP4 inhibitor prescription. (**A**) Patients prescribed any DPP4 inhibitor were divided into two groups, i.e., those who did and those who did not experience BP onset within the observation period. HbA1c values at the first and last measurements were compared before and after treatment, respectively. (**B**) Patients prescribed any DPP4 inhibitor were divided into two groups, i.e., those who received and those who did not receive lisinopril simultaneously. HbA1c values at the first and last measurements were compared before and after treatment, respectively. Statistical significance was determined using a two-tailed Wilcoxon matched-pair signed-rank test, ** $p < 0.01$; **** $p < 0.0001$. Statistical analysis for comparing HbA1c changes between groups was performed using the unpaired *t*-test with Welch's correction. n.s., not significant.

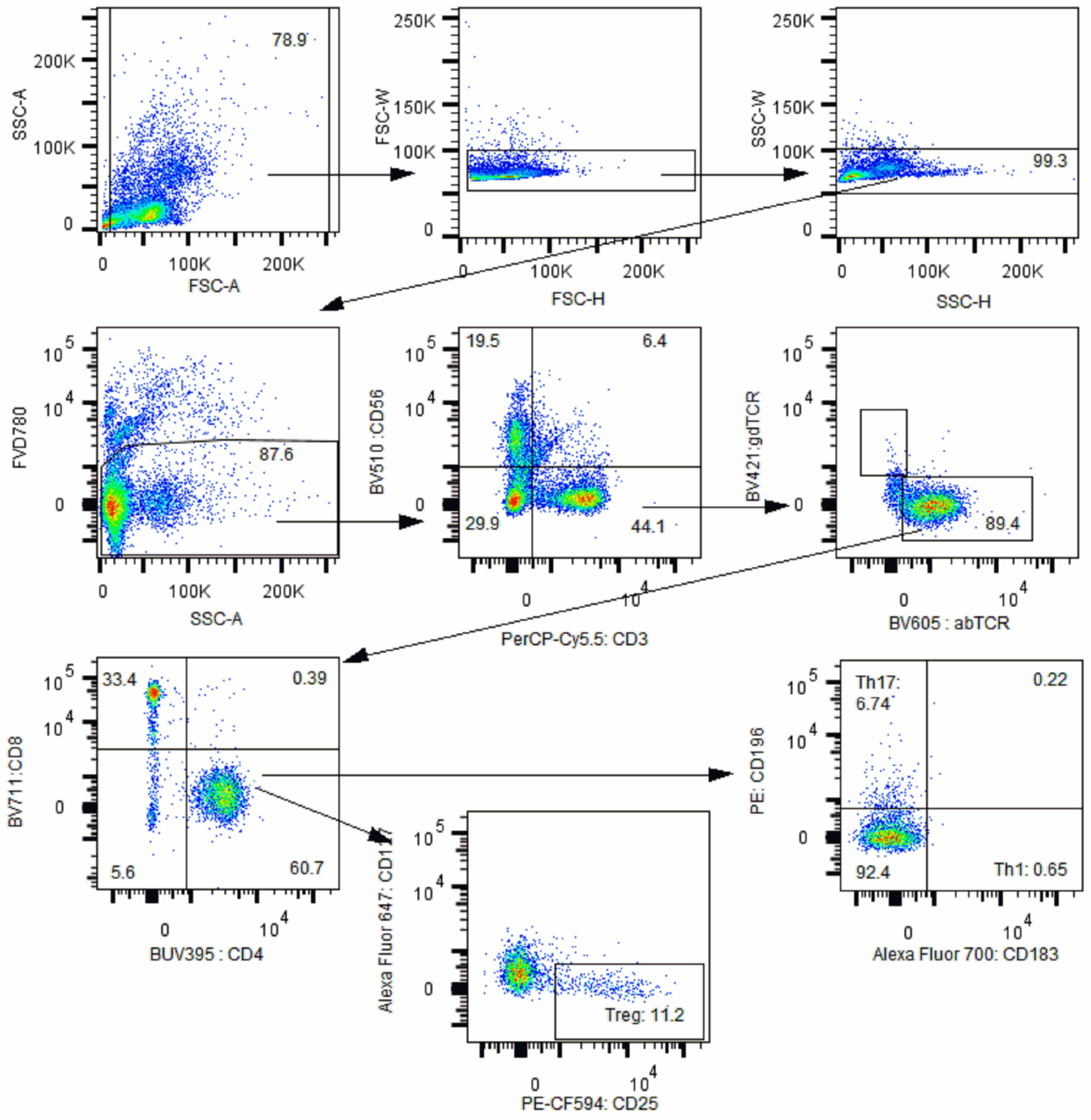


Supplemental Figure 3.

Gating strategy for T cell subsets using cell surface marker. Flow cytometry data corresponding to hPBMCs from healthy volunteers are shown as an example to demonstrate the gating strategy used to identify Th1, Th17, and Treg cells. Each cell type, including $CD4^+$ T cells ($CD3^+$ $CD56^-$ $\alpha\beta TCR^+$ $\gamma\delta TCR^-$ $CD8^-$ $CD4^+$), Th1 cells ($CD4^+$ T cells $CD196^-$ $CD183^+$), Th17 cells ($CD4^+$ T cells $CD196^+$ $CD183^-$), and Tregs ($CD4^+$ T cells $CD25^+$ $CD127^-$), was identified.

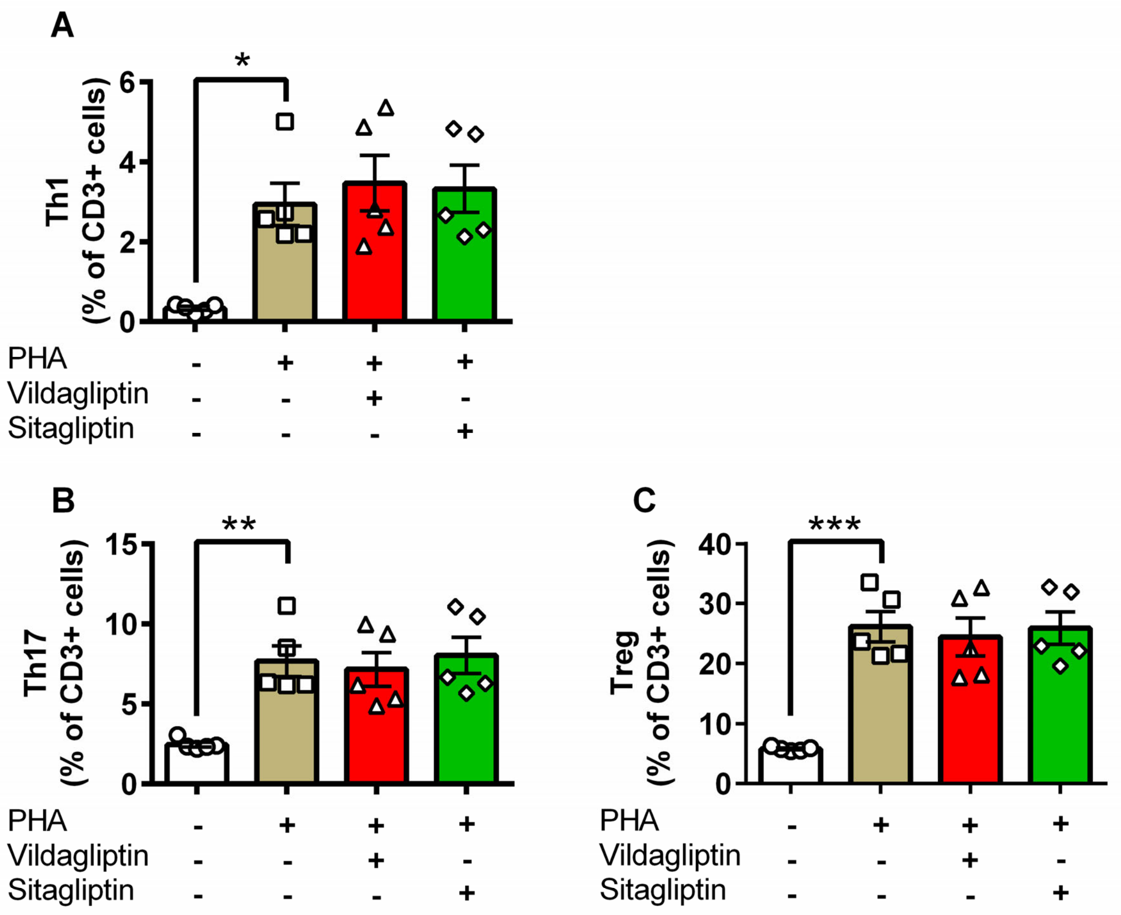


Supplemental Figure 4.

DPP4 inhibitors showing no effect on T cell phenotype. hPBMCs were stimulated with PHA with or without DPP4 inhibitors for 48 h. T cell subsets were characterized using cell surface markers (Supplemental Figure 3). (**A**) Percentage of Th1 cells among living $CD3^+$ cells. (**B**) Percentage of Th17 cells among living $CD3^+$ cells. (**C**) Percentage of Treg cells among living $CD3^+$ cells. Individual data are shown as the mean ± SEM. *$p < 0.05$, **$p < 0.01$, ***$p < 0.005$

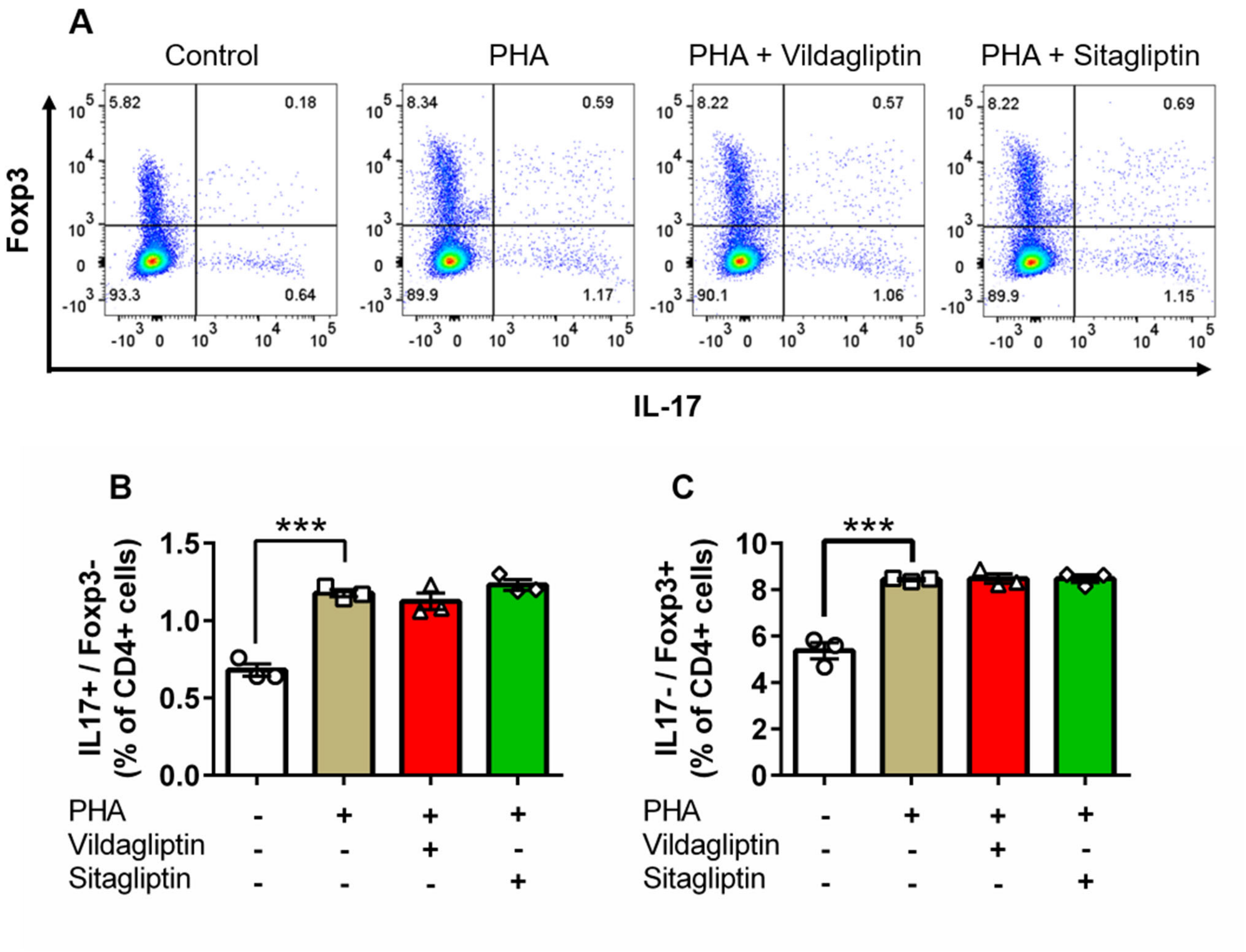


Supplemental Figure 5

T cell subsets characterized based on IL-17 and Foxp3 expression. (**A**) Dot plots representing each condition. (**B**) Percentage of IL-$17^{+}$/$Foxp3^{-}$ cells among living $CD4^{+}$ cells. (**C**) Percentage of $Foxp3^{+}$/IL-$17^{-}$ cells among living $CD4^{+}$ cells. Individual data are shown as the mean ± SEM. ***$p <$ 0.005.

Supplemental Table 1. Overall results of disproportionality analysis for BP in the FDA Adverse Event Reporting System (FAERS) database.

Filename: Supplemental Table 1.xlsx (contains 2 tables)

Sheet: BP_DrugA

Individuals in the FAERS database were divided into the following four groups: (*a*) individuals who received the drug of interest (drug A) and exhibited BP; (*b*) individuals who received drug A, but did not exhibit BP; (*c*) individuals who did not receive drug A and exhibited BP; and (*d*) individuals who did not receive drug A and did not exhibit BP. The reporting odds ratio (ROR) and 95% confidence interval (CI) as well as the *Z* score for DPP4 inhibitor-associated BP were calculated using the following respective equations:

$$\text{ROR} = \frac{a/b}{c/d}$$

$$95\%\ \text{CI} = exp\left\{\log(\text{ROR}) \pm 1.96\sqrt{\left(\frac{1}{a}+\frac{1}{b}+\frac{1}{c}+\frac{1}{d}\right)}\right\}$$

$$Z\ \text{score} = \frac{\log(\text{ROR})}{\sqrt{\left(\frac{1}{a}+\frac{1}{b}+\frac{1}{c}+\frac{1}{d}\right)}}$$

where *a*, *b*, *c*, and *d* represent the number of individuals in each group, respectively.

Sheet: DPP4iBP_DrugB

Individuals who received any DPP4 inhibitor were divided into the following four groups: (*a1*) individuals who received a concomitant drug of interest (drug B) and exhibited BP; (*b1*) individuals who received drug B, but did not exhibit BP; (*c1*) individuals who did not receive drug B and exhibited BP; and (*d1*) individuals who did not receive drug B and did not exhibit BP. The ROR and 95% CI as well as the *Z* score for DPP4 inhibitor-associated BP were calculated using the following respective equations:

$$\text{ROR} = \frac{a1/b1}{c1/d1}$$

$$95\%\ \text{CI} = exp\left\{\log(\text{ROR}) \pm 1.96\sqrt{\left(\frac{1}{a1}+\frac{1}{b1}+\frac{1}{c1}+\frac{1}{d1}\right)}\right\}$$

$$Z\ \text{score} = \frac{\log(\text{ROR})}{\sqrt{\left(\frac{1}{a1}+\frac{1}{b1}+\frac{1}{c1}+\frac{1}{d1}\right)}}$$

where *a1*, *b1*, *c1*, and *d1* refer to the number of individuals in each group, respectively.

Supplemental Table 2. Formulations of DPP4 inhibitors and lisinopril in the analysis of MarketScan data. The ingredients of formulations and the number of patients are shown.

| **Drug class** | **Formulations** | **Route** | **No. of patients** |
|---|---|---|---|
| DPP4 inhibitor | Sitagliptin Phosphate | Oral | 213,357 |
| | Metformin Hydrochloride/Sitagliptin Phosphate | Oral | 113,958 |
| | Linagliptin | Oral | 42,792 |
| | Empagliflozin/Linagliptin | Oral | 12,340 |
| | Saxagliptin Hydrochloride | Oral | 11,472 |
| | Linagliptin/Metformin Hydrochloride | Oral | 8,762 |
| | Metformin Hydrochloride/Saxagliptin Hydrochloride | Oral | 7,092 |
| | Alogliptin Benzoate | Oral | 3,468 |
| | Alogliptin Benzoate/Pioglitazone Hydrochloride | Oral | 1,503 |
| | Alogliptin Benzoate/Metformin Hydrochloride | Oral | 1,240 |
| | Dapagliflozin/Saxagliptin | Oral | 542 |
| | Ertugliflozin/Sitagliptin | Oral | 290 |
| Lisinopril | Lisinopril | Oral | 1,965,744 |
| | Hydrochlorothiazide/Lisinopril | Oral | 586,790 |

Supplemental Table 3. Definition of bullous pemphigoid in the analysis of MarketScan data. The number of patients for each ICD10 code is shown.

| ICD10 | Detail | No. of patients |
|---|---|---|
| L10 | (Non-Billable Dx) Pemphigus | 32 |
| L100 | Pemphigus vulgaris | 1,693 |
| L101 | Pemphigus vegetans | 101 |
| L102 | Pemphigus foliaceous | 338 |
| L103 | Brazilian pemphigus [fogo selvagem] | 18 |
| L104 | Pemphigus erythematosus | 103 |
| L105 | Drug-induced pemphigus | 21 |
| L1081 | Paraneoplastic pemphigus | 25 |
| L1089 | Other pemphigus | 323 |
| L109 | Pemphigus, unspecified | 1,258 |
| L12 | (Non-Billable Dx) Pemphigoid | 6 |
| L120 | Bullous pemphigoid | 3,326 |
| L121 | Cicatricial pemphigoid | 1,022 |
| L122 | Chronic bullous disease of childhood | 36 |
| L1230 | Acquired epidermolysis bullosa, unspecified | 184 |
| L1231 | Epidermolysis bullosa due to drug | 22 |
| L1235 | Other acquired epidermolysis bullosa | 57 |
| L128 | Other pemphigoid | 319 |
| L129 | Pemphigoid, unspecified | 729 |
| L270 | Generalized skin eruption due to drugs and medicaments taken internally | 55,717 |

Supplemental Table 4. Cell surface marker antibodies used during flow cytometry.

| **Antibody** | **Dilution** | **Fluorophore** | **Clone** | **Company** | **Catalog Number** |
|---|---|---|---|---|---|
| CCR6(CD196) | 1:50 | PE | 11A9 | BD Biosciences | 559562 |
| CD127 | 1:20 | Alexa Fluor® 647 | HIL-7R-M21 | BD Biosciences | 558598 |
| CD25 | 1:50 | PE-CF594 | M-A251 | BD Biosciences | 562403 |
| CD3 | 1:5 | PerCP-Cy5.5 | SK7 | BD Biosciences | 340949 |
| CD4 | 1:100 | BUV395 | RPA-T4 | BD Biosciences | 564724 |
| CD56 | 1:100 | BV510 | NCAM16.2 | BD Biosciences | 563041 |
| CD8 | 1:200 | BV711 | RPA-T8 | BD Biosciences | 563677 |
| CXCR3(CD183) | 1:50 | Alexa Fluor® 700 | 1C6/CXCR3 | BD Biosciences | 561320 |
| TCRαβ | 1:100 | BV605 | IP26 | Biolegend | 306732 |
| TCRγδ | 1:50 | BV421 | B1 | BD Biosciences | 562560 |
